# Cloning and Functional Characterization of Three Odorant Receptors From the Chinese Citrus fly *Bactrocera minax* (Diptera: Tephritidae)

**DOI:** 10.3389/fphys.2020.00246

**Published:** 2020-03-25

**Authors:** Yipeng Liu, Zhongyi Cui, Guirong Wang, Qiong Zhou, Yang Liu

**Affiliations:** ^1^College of Life Sciences, Hunan Normal University, Changsha, China; ^2^State Key Laboratory for Biology of Plant Diseases and Insect Pests, Institute of Plant Protection, Chinese Academy of Agricultural Sciences, Beijing, China; ^3^Guangdong Laboratory of Lingnan Modern Agriculture, Genome Analysis Laboratory of the Ministry of Agriculture, Agricultural Genomics Institute at Shenzhen, Chinese Academy of Agricultural Sciences, Shenzhen, China

**Keywords:** odorant receptor, *Bactrocera minax*, expression profile, *Xenopus* oocytes, 1-Octen-3-ol

## Abstract

Insect olfactory sensing is crucial for finding food, mating, and oviposition preference. Odorant receptors (ORs) play a central role in the transmission of odorant signals into the environment by the peripheral olfactory system. Therefore, the identification and functional study of ORs are essential to better understand olfactory mechanisms in insects. OR studies on Diptera insects are primarily performed on *Drosophila* and mosquitoes, but few studies have been reported in Tephritidae. In this study, we examined three candidate ORs (BminOR3, BminOR12, and BminOR16) from *Bactrocera minax*. Our analysis of tissue expression revealed that the three BminORs were expressed in the antennae, with no difference between the male and female. In *in vitro* heterologous expression system of *Xenopus* oocytes. BminOR3/BminOrco responded strongly to 1-octen-3-ol, BminOR12/BminOrco responded to eight compounds [methyl salicylate, benzaldehyde, (Z)-3-hexenyl acetate, butyl acrylate, butyl propionate, 1-octanol, (S)-(+)-carvone and benzyl alcohol], and BminOR16/BminOrco slightly responded to undecanol. Our results concluded that BminOR3, BimOR12, and BminOR16 could play an important role in host-finding and oviposition positioning in *B. minax*.

## Introduction

The insect’s olfactory system plays an important role in adaptation to the environment and survival, such as regulating the location of the insect’s host, oviposition, and predator avoidance (Bruce et al., 2005; Song et al., 2008). The olfactory sensillum on the antennae plays an important role in recognizing the odorant signal in insects. These olfactory sensilla are unique to the epidermal cells of the antennae, which are connected to the nervous system. This regulates the relationship between insect behaviors and the odorant signals in the environment. These hydrophobic molecules in the external environment enter into the sensory lymph and combine with odorant-binding proteins (OBPs) to form an odor molecule\odor binding protein complex, which finally reaches the surface of the dendritic membrane of the olfactory receptor neurons (ORNs). Either the molecules or the complex activates the corresponding ORs, which are then distributed on the dendritic membrane of the ORNs. This transmits the olfactory signal downstream, causing related behaviors in the insect (Leal, 2013).

The identification of thousands of odor molecules with different structures by insects primarily depends on the specific binding of various odor molecules by ORs, and an OR usually recognizes an odor molecule or a class of odor molecules (Shepherd and Shepherd, 1994; Clyne et al., 1999; Vosshall et al., 1999; Zwiebel and Takken, 2004). Detailed study of the structure and function of ORs is important for understanding the molecular mechanisms of olfactory recognition. Insect ORs have a topological structure that is diametrically opposed to vertebrate ORs, with the N-terminus of the receptor’s protein located in the cell membrane and the C-terminus located outside the cell membrane (Benton et al., 2006; Wistrand et al., 2006). In 1999, the first OR gene identified in an insect was found in *Drosophila melanogaster* (Clyne et al., 1999; Gao and Chess, 1999). Since then, the proliferation of bioinformatics has led to the identification of multiple insect ORs (Fox et al., 2001; Sakurai et al., 2004; Robertson and Wanner, 2006; Liu et al., 2012). Presently, OR function has been studied in several order of insects, such as Lepidoptera, Diptera, Hymenoptera, and Hemiptera (Kurtovic et al., 2007; Wanner et al., 2007; Zhang et al., 2016; Liu et al., 2018). Research on the function of OR in insects has led to a deeper understanding of the molecular mechanisms of olfactory recognition in mating, host location, and selection of oviposition sites. These provide new mechanisms for future pest control and can improve existing control strategies.

*Bactrocera minax* (Enderlein) is an international pest that is often the target of quarantine. Adults lay eggs in the fruit, the result of which can inflict significant economic losses on the citrus industry (Dorji et al., 2006; Drew et al., 2006). The larvae feed on the fruits, causing the fruit to fall off before maturation. They then enter the soil and pupate, which ensures they will return as a pest the next season. The other part of the fruits that have not been dropped by larvae are picked and sold along with healthy fruits, which are then spread to other areas. This results in an expansion of epidemic areas. These hidden larvae and pupa mean that *B. minax* is difficult to control, making the olfactory trapping of adults an important means of controlling the population of *B. minax* (Dong et al., 2014; Liu and Zhou, 2016). However, there are few studies on the molecular mechanism of olfactory recognition of *B. minax*. In this study, three general ORs (BminOR3, BminOR12, and BminOR16) were identified and cloned using data generated from transcriptomic analyses (unpublished). RT-PCR was used to demonstrate that three general ORs were highly expressed in the antennae of *B. minax*. By combining *in vitro* expression of *Xenopus* oocytes with the two-electrode voltage clamp technique, we found that BminOR3/BminOrco responded strongly to 1-octen-3-ol, BminOR12/BminOrco responded to eight compounds [methyl salicylate, benzaldehyde, (Z)-3-hexenyl acetate, butyl acrylate, butyl propionate, 1-octanol, (S)-(+)-carvone and benzyl alcohol], and BminOR16/BminOrco responded slightly to undecanol. We speculate that BminOR3, BminOR12, and BminOR16 were involved in the recognition of host plants and the selection of suitable oviposition sites by *B. minax*.

## Materials and Methods

### Insects Rearing and Tissues Collection

The fruit infested with *B. minax* larvae were collected from the citrus orchards of Lixian County (111.75E, 29.63N), Hunan Province, China, in September 2018. Pupation and eclosion occurred the next year, while the colony was maintained at 60 ± 5% relative humidity, 25 ± 1°C, and a 14:10 (L:D) photoperiod at the Institute of Plant Protection, Chinese Academy of Agriculture Sciences, China. The adults were fed brown sugar, yeast extract, and water in a ratio of 3:1:10. The antennae, head, abdomen, thorax, and legs were excised from 12- to 15 days old female and male adults, and immediately transferred to liquid nitrogen at −80°C until use.

### RNA Isolation and cDNA Synthesis

Total RNA was extracted from the antennae, heads, abdomens, thoraxes, and legs using Trizol Reagent (Invitrogen, Carlsbad, CA, United States). The RNA was quantified and assayed for purity using gel electrophoresis and NanoDrop ND-2000 Spectrophotometer (NanoDrop Technologies, Inc., Wilmington, DE, United States), and treated with DNaseI (TransGenBiotech, China) to remove trace amounts of genomic DNA. The first single-strand cDNA synthesis used RevertAid First Strand cDNA Synthesis Kit (Thermo Fisher Scientific, United States) and served as a template in PCR or RT-PCR reactions.

### Gene Cloning and Sequence Analysis

In the transcriptome of *B. minax*, unigenes were annotated using blastx against the NCBI non-redundant (nr) sequences with *e* < 1e-5. Candidate unigenes encoding putative BminORs were selected according to the annotation result. Based on the transcriptomes and RT-PCR results (unpublished), The sequences of the three BminORs (BminOR3, BminOR12 and BminOR16) were selected. The open-reading frames (ORFs) of the three BminORs were cloned using specific primers (Table 1). PCR reaction of 25 μL contained 12.5 μL 2 × PrimeSTAR Mix, 1 μL cDNA template, 1 μL of upstream and downstream primers (10 μM), ddH_2_O 9.5 μL. PCR reactions were performed under the following conditions: 98°C for 3 min; 35 cycles of 98°C for 10 s, 55°C for 15 s, 72°C for 90 s; 72°C for 10 min. PCR amplification products were purified by 1.0% agarose gels, and ligated with the pEASY-Blunt vector (TransGenBiotech, China), and sequenced by BGI (Beijing, China). Amino acid sequences (BminOR3, BminOR12 and BminOR16) along with 62 ORs from *D. melanogaster* (Clyne et al., 1999; Gao and Chess, 1999), 50 ORs from *Calliphora stygia* (Leitch et al., 2015), 85 ORs from *Musca domestica* (Scott et al., 2014), and 65 ORs from *Bactrocera dorsalis* (genome: assembly ASM78921v2) were used to construct a phylogenetic tree by RaxML version 8 with the Jones-Taylor-Thornton amino acid substitution model (JTT). Node support was assessed using a bootstrap method based on 1000 replicates (Cao et al., 2014).

**TABLE 1:** Primers’ sequence in this study.

**Primer name**	**Sequence (5′– 3′)**
**Specific primers for cloning**	
BminOR3F	G*ACTAGT*GCCACCATGTTATTCAATCCAAAACC GTTAAT (*Spe* I)
BminOR3R	ATTT*GCGGCCGC*TTAGTTTTCTTCGTTCTCGTA GAAACTT (*Not* I)
BminOR12F	G*ACTAGT*GCCACCATGATTTTGGAAAATGAGGAAG (*Spe* I)
BminOR12R	ATTT*GCGGCCGC*TCAATTCTCTTGAAAATT TTGC (*Not* I)
BminOR16F	G*ACTAGT*GCCACCATGACGCCATTATTCAA AAGCA (*Spe* I)
BminOR16R	ATTT*GCGGCCGC*TTAACCGTCCTGTTCCTC AATTC (*Not* I)
**Primers for RT-PCR**	
BminOR3RTF	CACCACGGAGAATGGCTGCAC
BminOR3RTR	GCCACAAGTGTAGGCGGCAAA
BminOR12RTF	TCTCATGCGGCATTTTGACAGC
BminOR12RTF	AACTGCACCCAAGCCGGAAA
BminOR16RTF	CCGAAGACGGAGGAGGAACGT
BminOR16RTR	TCGATACGCTCCGCATAATCCA
BminActinRTF	GAGAAGGGTCGTCGTATTCGTGAGT
BminActinRTR	CATTGTCGGGCAGTGGCTTCTT

**The restriction enzyme site added to each primer is indicated in parenthesis after the sequence, and the cutting sites are in italics.**

### Expression Profiles of the Three Odorant Receptors

Total RNA was extracted from the antennae (A), legs (L), heads without antennae (H), thoraxes (T), and abdomens (Ab) by Trizol Reagent (Invitrogen, Carlsbad, CA, United States). The first single-strand cDNA synthesis was performed according to the methods described above. The housekeeping gene BminActin (GenBank: MT130776) was used as control. Primers were designed using the Primer 5 software (PREMIER Biosoft International) and the sequences are available in Table 1. PCR reaction of 20 μL contained 8 μL ddH2O 10 μL 2 × EasyTaq PCR SuperMix (+ dye), 0.5 μL reverse primer (10 μM), 0.5 μL forward primer (10 μM), and 1 μL cDNA. PCR reactions were carried out under the following conditions: 94°C for 3 min; followed by 28 cycles of 94°C for 30 s, 55°C for 40 s, and 72°C for 60 s; then 72°C for 10 min. PCR amplification products were purified by 2.0% agarose gels. The experiment was repeated three times with independent RNA samples.

### Plant Volatile Compounds

The 44 plant volatiles used in the experiment were purchased from Sigma-Aldrich (purity ≥ 95%, Co. St. Louis, MO, United States), as shown in Table 2. First, prepare a 1M stock solutions in dimethyl sulfoxide (DMSO) and store in −20°C for use. Prior to the experiment, the stock solutions were diluted to a concentration of 10^–4^ M using a 1 × Ringer’s buffer (96 mM NaCl, 0.8 mM CaCl_2_, 2 mM KCl, 5 mM MgCl_2_ and 5 mM HEPES, pH 7.6). In order to ensure the reliability of the experimental results, the odor samples used in the experiments were formulated freshly.

**TABLE 2:** Test odorants in functional analysis of BminOR3, BminOR12, and BminOR16.

**No.**	**Name**	**No.**	**Name**
1	1-Hexanol	23	Nerolidol
2	1-Octen-3-ol	24	Ethyl butyrate
3	trans-2-Hexen-1-al	25	α-Humulene
4	Ethyl acetate	26	Nonanal
5	Methyl eugenol	27	(R)-(+)-Limonene
6	Hexanal	28	(S)-(−)-Limonene
7	Citral	29	Linalool
8	Myrcene	30	Butyl butyrate
9	Benzaldehyde	31	Butyl propionate
10	Methyl salicylate	32	Dibutyl ether
11	α-Pinene	33	2-Furaldehyde
12	(−)-trans-Pinocarveol	34	2-Methoxyphenol
13	β-Citronellol	35	Undecanol
14	Heptanal	36	Sabinene
15	(S)-(+)-Carvone	37	1-Octanol
16	Benzyl alchol	38	1-Nonanol
17	(−)-trans-Caryophyllene	39	p-Cymene
18	Isoamyl acetate	40	Butyl acrylate
19	Ocimene	41	(−)-β-Pinene
20	Farnesene	42	(−)-β-Elemene
21	α-Terpinene	43	(Z)-3-Hexenyl acetate
22	cis-3-Hexen-1-ol	44	Furfuryl alcohol

### Vector Construction and cRNA Synthesis

Primers with restriction enzyme cutting sites *Spe*I (GACTAGT), *Not*I (ATTTGCGGCCGC), and Kozak consensus sequences (GCCACC) were designed to amplify the ORFs of the BminOR3, BminOR12, and BminOR16. These were then ligated into pT7Ts with the same restriction enzyme cutting sites (*Spe*I and *Not*I) (Wang et al., 2011). After proper sequencing, the recombinant plasmid was extracted and linearized by *Eco*RI (GAATTC). The synthesis of cRNA was performed using mMESSAGE mMACHINE T7 kit (Ambion, Austin, TX, United States). The BminOR3, BminOR12, and BminOR16 cRNA were diluted to a concentration of 2 μg/μL with nuclease-free water and stored at −80°C.

### Receptor Expression in *Xenopus* Oocytes and Two Electrode Voltage-Clamp Electrophysiological Recordings

Subsequent electrophysiological recordings were performed according to previously reported protocols (Tan et al., 2007; Wang et al., 2010). Mature healthy *Xenopus* oocytes (stages V–VII) were separated and then incubated with a washing buffer containing 2 mg/mL collagenase for 45 min at 28°C, after which 27.6 ng BminOrco cRNA and 27.6 ng BminORs (BminOR3, BminOR12, and BminOR16) cRNA were microinjected (Wang et al., 2011). The injected oocytes were incubated in 1 × Ringer’s solution supplemented with 550 mg/mL sodium pyruvate, 5% dialyzed horse serum, 50 mg/mL tetracycline and 100 mg/mL streptomycin for 2–4 days at 16°C. Whole-cell currents were recorded from the injected oocytes with a two-electrode voltage clamp. Odorant-induced currents were recorded with an OC-725C oocyte clamp (Warner Instruments, Hamden, CT, United States) at holding potential of −80 mV. Oocytes were exposed to compounds in ascending order of concentration with an interval between exposures that allowed the current to return to baseline. The Digidata 1440A and pCLAMP 10.2 software (Axon Instruments Inc., Union City, CA, United States) were used to acquire and analyze all data. Dose-response data were analyzed using GraphPad Prism 5 (GraphPad Software Inc., San Diego, CA, United States).

## Results

### Gene Cloning and Sequences Analysis

**FIGURE 1:**
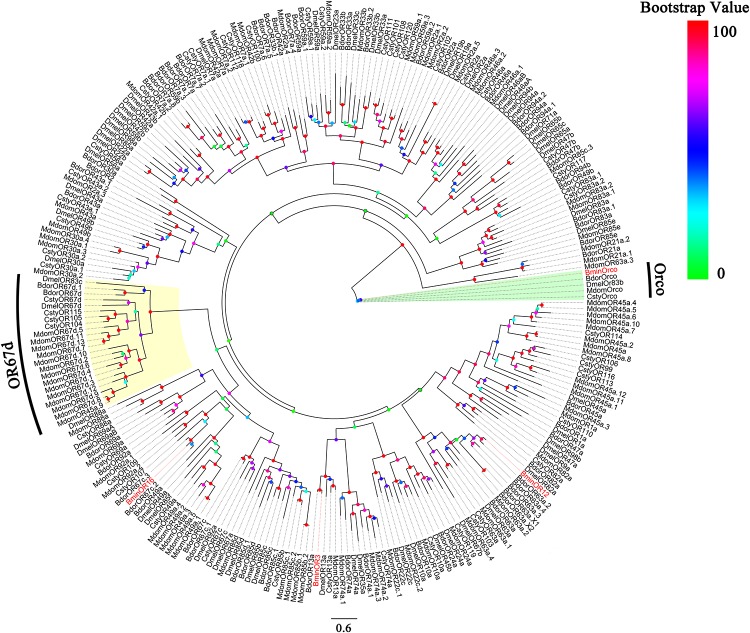
Phylogenetic analysis of the three candidate ORs with candidate ORs from Dipteran. Bmin, *B*. *minax*; Csty, *C*. *stygia*; Mdom, *M. domestica*; Dmel, *D*. *melanogaster*; Bdor, *B*. *dorsalis*. The clade in green indicates the Orco co-receptor gene clade and the one in yellow is the pheromone receptor gene clade. The three BminORs are in red.

**FIGURE 2:**
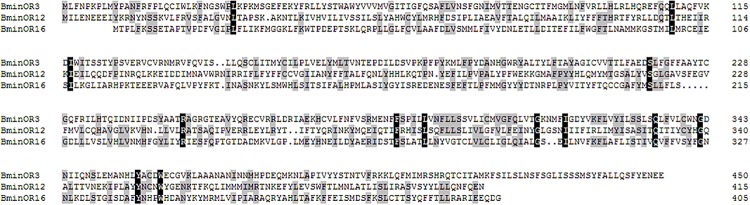
Alignment of the amino acid sequence of BminOR3, BminOR12, and BminOR16.

Based on the analysis of the *B. minax* transcriptome (unpublished), we cloned BminR3, BminOR12, and BminOR16 [GenBank: MN537976 (OR3), MN855530 (OR12) and MN537977 (OR16)] which contain complete open reading frames of 1353, 1248, and 1215 bp. These encode 450, 415, and 404 amino acid residues, respectively. The transmembrane domain analysis was performed on three BminORs, and the results showed that all three BminORs possess 6–7 putative transmembrane domains with extracellular C-terminus and intracellular N-terminus. This was contrary to the classic G-protein-coupled receptors. The phylogenetic tree was rooted by the Orco clade since it is considered a conserved OR subgroup different from conventional ORs. The clade of the pheromone receptors was detected since these ORs were closely clustered with DmelOR67d, the well-known pheromone receptor in *D. melanogaster*. BminOR3, BminOR12, and BminOR16 were distinctly separated from other Diptera sex pheromone receptors, and clustered with general ORs, which proved that BminOR3, BminOR12, and BminOR16 are general ORs of *B. minax* (Figure 1). These three ORs exhibited high diversity in their protein sequence, since the similarity among them is only 22% (Figure 2). This suggests they might have different functions.

### Expression Profiles of the Three ORs

**FIGURE 3:**
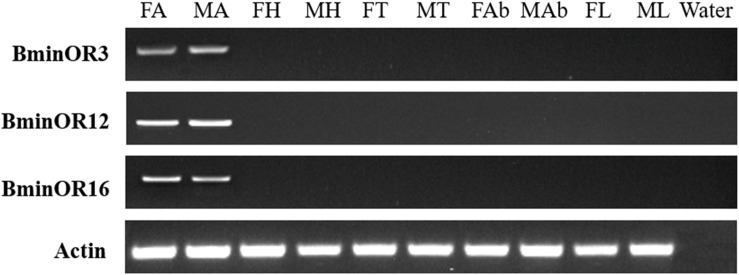
Expression profiles of BminOR3, BminOR12, and BminOR16 in different tissues of male and female *B. minax*. FA, female antennae; MA, male antennae; FH, female heads (without antennae); MH, male heads (without antennae); FT, female thoraxes; MT, male thoraxes; FAb, female abdomens; MAb, male abdomens; FL, female legs; ML, male legs.

RT-PCR analysis of BminOR expression showed that BminOR3, BminOR12, and BminOR16 were expressed in the antennae, since their expression was not detected in the head, thorax, abdomen, and leg tissues. The expression of all three BminORs shows no difference between male and female. Among these, the expression levels of BminOR12 and BminOR16 are higher than that of BminOR3 in both the male and female antennae (Figure 3).

### Functional Characterization of the Three ORs

**FIGURE 4:**
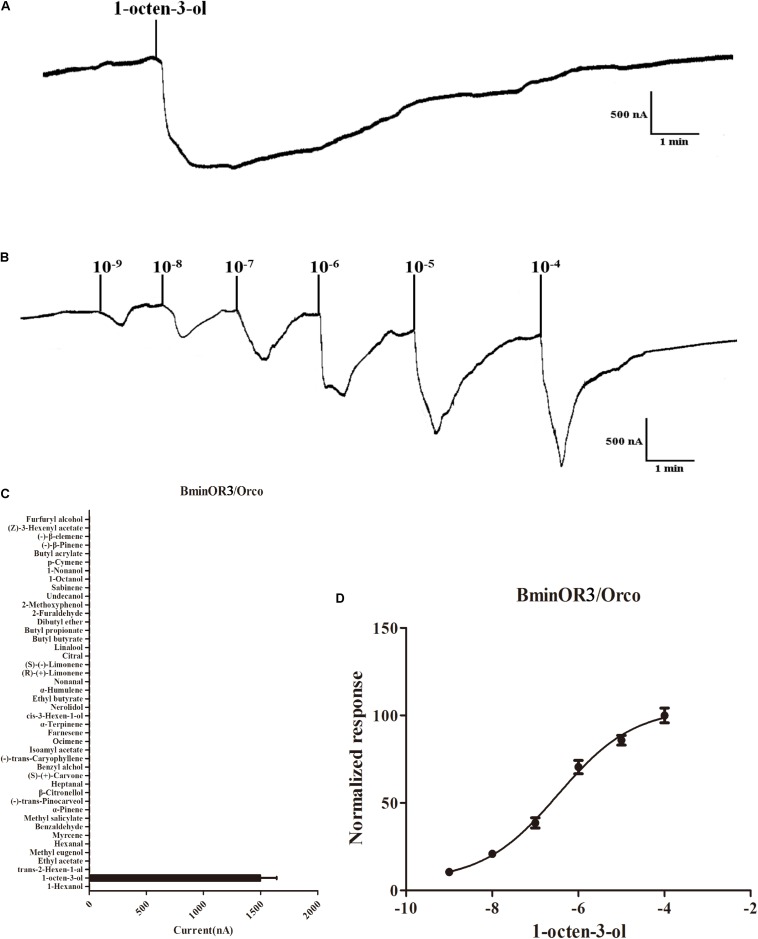
Responses of *Xenopus* oocytes with co-expressed BminOR3/BminOrco to stimulation with volatile compounds. **(A)** Inward current responses of BminOR3/BminOrco *Xenopus* oocytes in response to 10^–4^ M of the volatile compounds. **(B)** BminOR3/BminOrco *Xenopus* oocytes stimulated with a range of 1-octen-3-ol concentrations. **(C)** Response profile of BminOR3/BminOrco *Xenopus* oocytes. Error bars indicate SEM (*n* = 6) (*T*-test, *P* < 0.05). **(D)** Dose-response curve of BminOR3/BminOrco *Xenopus* oocytes to 1-octen-3-ol. 1-Octen-3-ol EC50 = 3.467 × 10^–7^ M. Error bars indicates SEM (*n* = 6).

BminOR3, BminOR12, and BminOR16 were co-expressed with the BminOrco (GenBank: MT130775) in *Xenopus* oocyte, and functionally characterized using the voltage clamp recording system. Our results showed that the oocytes expressed in the three BminORs are activated by at least one of the 44 plant volatiles at a concentration of 10^–4^ M. They also exhibited different response profiles. BminOR3/BminOrco showed strong responses to 1-octen-3-ol, where the mean response value was 1496 nA. It showed no response to all the other test plant volatiles (Figures 4A,C). In dose-response studies, we assayed the responses of BminOR3/BminOrco to a range of concentrations of 1-octen-3-ol. The response of BminOR3/BminOrco to 1-octen-3-ol is very sensitive, even the lowest concentration (10^–9^ M) can elicit measurable responses. The calculated half maximal effective concentration (EC50) value was 3.391 × 10^–7^ M (Figures 4B,D).

**FIGURE 5:**
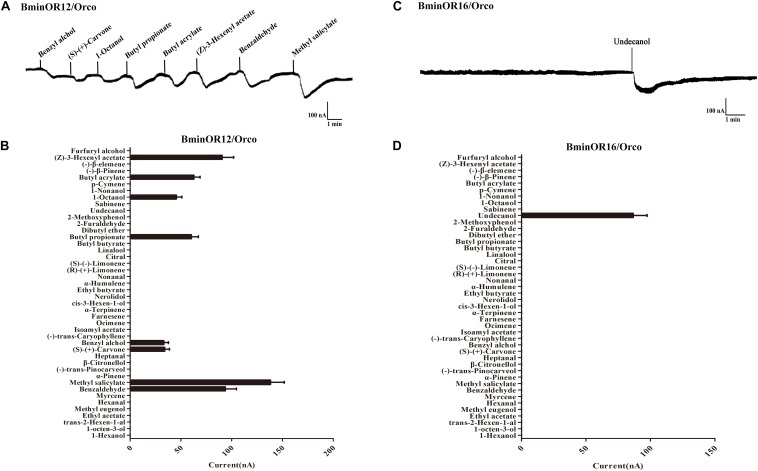
Responses of *Xenopus* oocytes with co-expressed BminOR12/BminOrco and BminOR16/BminOrco to stimulation with volatile compounds. Inward current responses of BminOR12/BminOrco **(A)** and BminOR16/BminOrco **(C)** *Xenopus* oocytes in response to 10^–4^ M of the volatile compounds. Response profile of BminOR12/BminOrco **(B)** and BminOR16/BminOrco **(D)** *Xenopus* oocytes. Error bars indicate SEM (*n* = 6) (*T*-test, *P* < 0.05).

BminOR12/BminOrco responded to eight compounds: benzyl alcohol, (S)-(+)-carvone, 1-octanol, butyl propionate, butyl acrylate, (Z)-3-hexenyl acetate, benzaldehyde, and methyl salicylate. The respective mean response values of these are 33, 34, 46, 60, 63, 91, 94, and 138 nA at a concentration of 10^–4^ M (Figures 5A,B). Under the same conditions, BminOR16/BminOrco responded weakly to undecanol, with a mean response value of 87 nA (Figures 5C,D). The responses of the two ORs against the compounds at lower concentrations were too small to be detected, and we were not able to obtain their EC50 to different ligands.

## Discussion

Insects recognize different odor molecules in the environment by way of a complex and sophisticated olfactory system, which regulates their habitat selection, food finding, mating, reproduction, clustering, avoidance, and information transmission (Hansson and Stensmyr, 2011; Gadenne et al., 2016). Previous studies have reported that ORs serve an important role in the olfactory recognition systems in insects (Leal, 2013; Wicher, 2014; Bohbot and Pitts, 2015). Based on the transcriptome analysis of *B. minax* (unpublished), we identified three BminORs (BminOR3, BminOR12, and BminOR16) from the antennae of *B. minax* and obtained the full-length sequences. Presently, studying the expression pattern of ORs in different tissues explores OR function. We found no significant difference in OR expression between male and female antennae, while there were some ORs with high or specific expression in the female or male antennae (Krieger et al., 2002; Tanaka et al., 2009; Liu et al., 2014; Zhang et al., 2016). RT-PCR analysis revealed that BminOR3, BminOR12, and BminOR16 were specifically expressed in both male and female antennae. This indicates that these BminORs could be related to the identification of the host plant.

We further analyzed the function of BminOR using *Xenopus*-expression. BminOR3 responded robustly to 1-octen-3-ol, BminOR12 responded to eight compounds [methyl salicylate, benzaldehyde, (Z)-3-hexenyl acetate, butyl acrylate, butyl propionate, 1-octanol, (S)-(+)-carvone and benzyl alcohol], and BminOR16 slightly responded to undecanol. In *B. dorsalis*, BdorOR13a responded robustly to 1-octen-3-ol in the *Xenopus* oocytes recording system, which could enhance landing behavior in mated females (Miyazaki et al., 2018). BminOR3 clustered with BdorOR13a in the phylogenetic tree, while the homolog of BminOR3 in *B. dorsalis*, BdorOR13a, shared an amino acid similarity of 85.59% with BminOR3. This suggests that 1-octen-3-ol could exhibit an attractive effect on *B. minax*. Meanwhile, the EC50 value of 1-octen-3-ol was 3.467 × 10^–7^ and showed similar sensitivity with EC50 of moth PRs to sex pheromones (Nakagawa et al., 2005; Wanner et al., 2010; Wang et al., 2011), This suggests that 1-octen-3-ol may be important for *B. minax*. BminOR12 co-expressed with BminOrco responded to eight compounds. Similar studies on the function of ORs have been performed on other insects (Di et al., 2017; Fouchier et al., 2017; Zhang et al., 2019). Of these eight compounds, methyl salicylate not only elicited higher antennal response, but is also an important component of attractants in *Anastrepha ludens* (Rasgado et al., 2009). Additionally, (Z)-3-hexenyl acetate can evoke EAG responses and attractive effects on both sexes of *Dacus ciliates* (Alagarmalai et al., 2009). This suggests that methyl salicylate and (Z)-3-hexenyl acetate could also have an attractive effect on *B. minax*. BminOR16 co-expressed with BminOrco slightly responded to undecanol, while there may be another BminOR that responds to undercanol. Previously, we studied the oviposition preference of *B. minax*, with results showing that females prefer oviposition in the basal hemisphere of Shatian you (*Citrus maxima*) and Amakusa, and GC-MS analysis showing that the volatiles in the basal hemisphere of Shatian you (*Citrus maxima*) and Amakusa contain undecanol (Liu, 2015). This indicated that BminOR16 is involved in determining oviposition in *B. minax*. BminOR3 and BminOR16 belong to narrowly tuned receptors, which are thought to carry biologically relevant information (Wilson and Mainen, 2006; Carey et al., 2010). Altogether, BminOR3, BminOR12, and BminOR16 play irreplaceable roles in host location and selection of oviposition sites by *B. minax*. At the same time, *B. minax* reproduces once each year, which makes *in vivo* experiments difficult. In order to better understand olfactory recognition in *B. minax*, a focus on molecular experiments is in order.

## Conclusion

In conclusion, we have identified and studied the functions of three general ORs (BminOR3, BminOR12, and BminOR16) of *B. minax.* The specific functions of ligands (1-octen-3-ol, methyl salicylate, and undecanol) on *B. minax* required to be further studied, especially in the behavioral experiments. Further study of ORs, along with previous studies in chemical ecology, can help explain the molecular mechanisms of insect feeding, oviposition, and mating. They also provide a theoretical basis for the development of high-efficiency repellents as well as novel methods of pest control.

## Data Availability Statement

The datasets generated for this study can be found in the article/supplementary material. The cDNA sequences of BminR3, BminOR12, BminOR16, BminOrco, and BminActin can be found in Genbank with the accession numbers MN537976, MN855530, MN537977, MT130775, and MT130776.

## Author Contributions

YiL, ZC, GW, YaL, and QZ conceived and designed the experiments. YiL and ZC performed the experiments and analyzed the data. YiL, ZC, and YaL contributed reagents, materials, and analysis tools. YiL, GW, YaL, and QZ wrote the manuscript. All authors contributed to research design and manuscript preparation, and read and approved the final manuscript.

## Conflict of Interest

The authors declare that the research was conducted in the absence of any commercial or financial relationships that could be construed as a potential conflict of interest.
